# Action Possibility Judgments of People with Varying Motor Abilities Due to Spinal Cord Injury

**DOI:** 10.1371/journal.pone.0110250

**Published:** 2014-10-31

**Authors:** Gerome A. Manson, Dimitry G. Sayenko, Kei Masani, Rachel Goodman, Lokman Wong, Milos R. Popovic, Luc Tremblay, Timothy N. Welsh

**Affiliations:** 1 Faculty of Kinesiology & Physical Education, University of Toronto, Toronto, Ontario, Canada; 2 Centre for Motor Control, University of Toronto, Toronto, Ontario, Canada; 3 Department of Integrative Biology and Physiology, University of California Los Angeles, Los Angeles, California, United States of America; 4 Toronto Rehabilitation Institute, University Health Network, Toronto, Ontario, Canada; 5 Institute of Biomaterials and Biomedical Engineering, University of Toronto, Toronto, Ontario, Canada; University G. d'Annunzio, Italy

## Abstract

Predictions about one's own action capabilities as well as the action capabilities of others are thought to be based on a simulation process involving linked perceptual and motor networks. Given the central role of motor experience in the formation of these networks, one's present motor capabilities are thought to be the basis of their perceptual judgments about actions. However, it remains unknown whether the ability to form these action possibility judgments is affected by performance related changes in the motor system. To determine if judgments of action capabilities are affected by long-term changes in one's own motor capabilities, participants with different degrees of upper-limb function due to their level (cervical vs. below cervical) of spinal cord injury (SCI) were tested on a perceptual-motor judgment task. Participants observed apparent motion videos of reciprocal aiming movements with varying levels of difficulty. For each movement, participants determined the shortest movement time (MT) at which they themselves and a young adult could perform the task while maintaining accuracy. Participants also performed the task. Analyses of MTs revealed that perceptual judgments for participant's own movement capabilities were consistent with their actual performance- people with cervical SCI had longer judged and actual MTs than people with below cervical SCI. However, there were no between-group differences in judged MTs for the young adult. Although it is unclear how the judgments were adjusted (altered simulation vs. threshold modification), the data reveal that people with different motor capabilities due to SCI are not completely biased by their present capabilities and can effectively adjust their judgments to estimate the actions of others.

## Introduction

Before engaging in a task (e.g., lifting a heavy object), it is important to be able to predict whether or not you are capable of successfully completing the task. Likewise, it is also important to predict whether another person, with different characteristics and abilities, can perform a task before engaging in joint motor action (e.g., imagine passing the heavy object to a child). Recent studies [1], [2] examining such action possibility judgments have indicated that the core of these judgments is a simulation process that utilizes linked action and perception networks (see [3] and [4] for in-depth discussions of possible mechanisms). Specifically, it has been proposed that when an individual is asked to make judgments about their own actions or the actions of another person, the individual first simulates the to-be-judged action. This simulation process is thought to involve the sub-threshold activation of the motor networks associated with the generation of the action and the perceptual networks associated with the action's consequences. After engaging in this simulation, the observer gains information about their own performance and uses this to form a prediction or judgment. To make a prediction for another person, it is thought that the individual, as before, simulates their own performance and subsequently adds or subtracts a correction factor based on an estimation of how different the other person's perceived motor capabilities are from their own [5]. Given the role of one's own motor capabilities in the formation and plasticity of the central networks involved in the simulation, it is hypothesized that one's capabilities may influence the formation of these action possibility judgments. The present study was designed to test if changes in motor capabilities affect an individual's ability to form accurate action possibility judgments of themselves and others.

Evidence supporting the role of action simulation in the formation of action possibility judgments has been drawn from studies examining perceptual judgments of upper-limb aiming movements. These judgment tasks have been employed because Fitts's Law [6] accurately characterizes the relationship between movement time (MT) and movement difficulty. Specifically, Fitts's Law predicts that as the difficulty of a movement increases, performers must increase their MT to maintain a high level of endpoint accuracy. This relationship between movement difficulty and MT is captured by the Fitts's Law equation: MT = a+b (log_2_ [2A/W]), where “a” and “b” are constants that relate the individual's base MT (y-intercept) and the change in MT for a given change in movement difficulty (slope of the regression line), respectively. The “(log_2_ [2A/W])” component of the equation quantifies the difficulty of the movement in bits (see [6]) of information. This Index of Difficulty (ID) is a function of the width of the target (W) and the movement amplitude (A). Effectively, ID, and as a result MT, increases as the width of the target decreases and/or the movement amplitude increases.

The first experiment to employ the Fitts's Law task to examine the processes of action possibility judgments was conducted by Grosjean [2]. Participants in this study were shown apparent motion displays of a human arm moving cyclically between two targets. To display movement contexts of varying IDs, the experimenters manipulated the target width and the distance between the targets across trials. The rate at which the hand moved between the two targets (apparent MT) was also varied across trials and participants were asked to judge whether or not it was possible to maintain accuracy while moving between the two targets at the displayed MT. Analysis of the MTs revealed the Fitts's Law relationship (i.e., MTs that were judged as the shortest possible MTs at which one could maintain accuracy increased as the IDs increased). The authors concluded that the appearance of the Fitts's Law relationship occurred because participants effectively simulated their own performance.

The hypothesis that a simulation process underlies action perception (i.e., the formation of these judgments) leads to numerous distinct predictions about: 1) a central role for the motor system during the formation of action possibility judgments (see [7] for fMRI evidence supporting this prediction); and, 2) the possible role an individual's own movement capabilities plays in shaping these judgments. Support for the latter prediction in the neuro-typical population has accumulated through a series of studies building on the work of Grosjean et al. [2]. In one set of studies, Chandrasekharan et al. [1] (see also [8]) investigated how recent task experience and the current body state of the participant influenced perceived MTs. It was predicted that if perception-action linkages and movement capabilities shaped action possibility judgments, then recent experience with the to-be-performed task and alterations in the observer's current body state should affect the judged MTs. The main task for these studies was based on the methods from the Grosjean et al. [2]. To assess the influence of task experience, participants completed the perceptual judgment tasks before and after actually performing the aiming task. Analysis of the MTs revealed that participants initially underestimated their capabilities as MTs in the pre-execution judgment task were significantly longer than the actual MTs in the execution task. In contrast, MTs from the post-execution judgment task were significantly shorter than MTs from the pre-execution task and, not different from actual MTs. These findings are consistent with the prediction that recent task experience and knowledge of their own motor capabilities would increase the accuracy of the simulation process and, as a result, the judgment process.

In a subsequent experiment, Chandrasekharan et al. [1] assessed the influence of current movement capabilities on the above-mentioned action possibility judgments. Participants completed the Fitts's Law perceptual judgment protocol twice: once with weights attached to their wrists and once without weights. Note that the weights were completely incidental to the judgment task in that the participants never actually performed the aiming task with or without weights. Consistent with the hypothesis that both the simulation and judgment processes are influenced by current movement capabilities, the judgment of the MTs made while wearing weights were longer than those made when participants were not wearing weights (see also [9]–[11]). The results of this study suggest that an individual's judgment of their own and others abilities can be influenced by incidental and temporary changes in motor capabilities.

Neuropsychological evidence also supports the ideas of a relationship between an individual's motor performance and the formation action possibility judgments. In one study, researchers examined the effects of neurological injury on action perception by observing how a person with a frontal brain lesion (damage to the left, inferior, middle, and superior gyri) resulting from a stroke would complete the Fitts's Law judgment task [12]. The judged MTs were compared to the person's actual performance MTs and to the MTs of control participants. Consistent with previous studies, the control group demonstrated Fitts's Law relationships in both the judgment and execution tasks. In contrast, the MTs of the person with the frontal lesion were not affected by target size in both the judgment and execution tasks. That is, MTs in both tasks increased as movement amplitude increased, but did not increase when the target width decreased. The findings of this study generated some initial support to the idea that changes in motor capabilities alter the one's action perception and judgment abilities. Additional work in this area is needed, however, because is it not exactly clear what lead to the observed patterns of MTs in the individual with the frontal lesion. For instance, it is possible that the participant was not able to perceive differences in target width because, as the authors of the study noted, lesions in this area of the brain are associated with misperceptions in context. Second, it is not known whether the participant was aware of this deficit. Thus, either misrepresentations in context or an altered simulation process could explain the results reported by this study. Overall, the literature presented above support the idea of a link between actions and judgments (see also [8]; cf. [13]).

The present investigation was designed to extend the understanding of the relationship between motor performance and action possibility judgments by assessing the relationship between these processes in people with varying motor capabilities as a result of spinal cord injury (SCI). This population was of theoretical interest because the higher-order perceptual and motor networks (i.e., cortical and subcortical centers of the brain) are intact, but actual motor function varies with the level of injury. Two groups of individuals were recruited. The main experimental group consisted of individuals with impaired or “atypical” upper-limb function that resulted from damage to the cervical region of the spinal cord. The second group consisted of people with damage to the spinal cord below the cervical region and, thus, had impaired functioning of the lower limbs, but had intact or “typical” upper-limb function. This second group was included in the design to determine if any potential performance differences in the tasks were due limb-specific changes in function and to control for other potential factors associated with SCI.

All participants were asked to make action possibility judgments for their own performance (self-judgment task) and for those of the college-aged male (approximately 24 years old and without any neurological disorders) depicted in the apparent motion displays (other-judgment task). Participants also completed the aiming task (execution task). It was predicted that there would be differences between the two groups in their actual performance MTs. People with cervical SCI (cervical group) were expected to have longer actual (execution) MTs than participants with below cervical SCI (below cervical group). This result was anticipated because the participants with cervical level injuries possess impaired upper-limb function. Thus, the comparisons of greater theoretical interest involved the MTs on the two judgment tasks. If the participants relatively accurately simulate their own performance when forming judgments about their own action possibilities, then the MTs in the “self” judgment task for the group with cervical damage should be longer than those for the group with below cervical SCI. Finally, the analysis of greatest theoretical relevance was the between-group comparison of MTs judged for the other person to perform. If the simulation of the person's own performance biases the judgments for what other people are able to perform, then the MTs for the cervical group in the “other” condition will also be higher than those for the group with below cervical SCI. On the other hand, if the participants with impaired upper-limb function are able to accurately account for differences between themselves and a neuro-typical individual (i.e., are not biased by their own capabilities), then there will be no between-group differences in MTs in the “other” condition.

## Materials and Methods

### Participants

Fourteen people with below cervical level (thoracic-lumbar-sacral) and eight people with higher level (cervical) SCI volunteered to take part in the study. Prior to participation, participants provided informed consent and answered a brief questionnaire where information about their injury, including the level, duration (number of years since injury), and their perceived upper arm function was recorded (for scale of perceived upper-limb ratings see Table 1). Only volunteers with some degree of upper-limb function completed the study due to the requirement to be able to perform the motor task. It is also important to note that the main categorical variable used to group participants was the level of SCI. Participants were given a $20 monetary compensation after completion of the experiment and all experimental procedures and protocols were approved by the Toronto Rehabilitation Institute Ethics Review Board. Details of the characteristics of the participants are displayed in Table 2.

**Table 1:** Subjective Report of Arm Function.

Classification	Characteristics of Report
1) Below functional	Person states that they can perform some movement with difficulty and can possibly complete the task
2) Functional	Person states that they can perform some movement with some difficulty and can complete the task
3) Good	Person states that arm is used regularly for daily activities with little difficulty
4) Very Good	Person states that arm is use regularly for daily activity with no difficulty

The table above shows the classification of arm function based on each participant's self-report.

**Table 2:** Demographic data of the participants.

Group	Age	Gender	Handedness	Level of injury	Time after injury	Self-report of arm function
Cervical	42	Male	Right	C5	28	Functional
	39	Male	Right	C4/C5	16	Functional
	51	Female	Right	C5/C6	19	Functional
	67	Female	Right	C3/C4	19	Functional
	50	Female	Right	C5	11	Good
	51	Female	Right	C2, C5/C6	4	Functional
	28	Male	Right	Spina Bifida	28	Functional
	37	Male	Right	C6/C7	16	Below Functional
Below Cervical	74	Male	Right	T9	12	Good
	70	Female	Right	T9/T10	5	Good
	56	Male	Right	T12-L2	32	Good
	47	Female	Right	T9, L1	28	Good
	32	Female	Left	L1	32	Good
	57	Male	Right	T9	12	Good
	45	Male	Right	Cocyx	5	Good
	60	Male	Right	T12	10	Good
	33	Female	Right	T9-T11	21	Good
	50	Male	Right	T10	7	Very Good
	67	Female	Right	Cocyx	5	Good

The demographic data obtained for each participant in each group.

### Apparatus, Stimuli, and Tasks

The design of the tasks in the present study was based on the methods of the aforementioned Fitts's Law experiments [2], [5]. Nine target contexts were built on 57×72.5 cm white poster boards. Each of the displays had two target locations (black strips of paper 15 cm in height) with a specific combination of target width (2, 4 or 8 cm) and distance (2, 4, 16, 32 or 64 cm center-to-center) to generate three displays with the IDs of 2, 3, and 4 [6].

**Figure 1:**
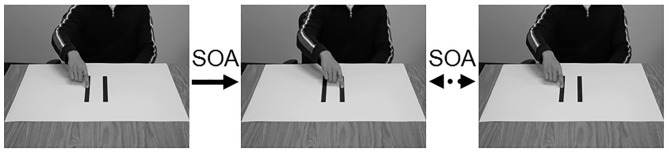
Right/Left images and sequence of events used for the apparent motion stimuli during the action perception task. The first arrow indicates that the first image was of the model with their finger on the right target and that it was followed by the image with model's finger on the left target presented after the specific SOA for that trial. The two-way arrow indicates that the images were alternately presented until the the enter key was pressed.

The stimuli for the action possibility judgment task were photographs of an adult male sitting in front of posters with the index finger of the right hand placed in the middle of one of two targets. Two photos, one with the finger on the right target and one with the finger on the left target, were taken for each poster board. The two pictures were alternated to create an apparent motion of the model moving the finger between the two targets (see Figure 1). The same pairs of pictures were displayed throughout a single trial so that the ID was consistent within a trial. The time between the presentations of the two pictures (the stimulus onset asynchrony [SOA]) served as the apparent MT for the judgment tasks.

The apparent motion stimuli were presented to participants on a 19-inch CRT monitor (ViewSonic, Graphics Series – G225f) running at a resolution of 1204×768 pixels and a refresh rate of 100 Hz. The monitor was placed at eye level, approximately 60 cm from the participant. There were two trials for each of the judgment conditions, one in which the SOA at the beginning of each trial was 50 and another in which the SOA at the beginning was 900 ms. Participants instructed the experimenter to increase or decrease the SOA (apparent MT) until the participant identified the shortest MT at which they judged it as possible to move and maintain accuracy. Instructions were given to the experimenter as it was anticipated that some participants with cervical injuries may experience difficulty using a standard keyboard. The length of the apparent MT was altered by pressing the cursor keys [8], [12]. A single press of the left and right arrow keys decreased and increased the apparent MT by 100 ms, respectively. To increase the resolution of the measurement, the down and up arrow keys increased and decreased the apparent MTs by 10 ms increments, respectively. When participants identified the shortest MT at which they judged it was possible to move and maintain accuracy, they told the experimenter to press the “Enter” key, at which point the SOA was recorded. After the SOA was recorded, the next trial commenced and a new set of pictures were displayed. A custom program written in Matlab (The Mathworks Inc.) controlled the presentation of the stimuli and the recording of the final SOA.

Participants completed the task on each of the nine combinations of target width and distance two times for each experimental condition (self and other). In the self-judgment condition, participants were asked to “estimate the lowest time required for *you* to complete the movements accurately”. In other-judgment condition, participants were instructed to “estimate the lowest time required for the *person in the video* to complete the movements accurately”. Participants were told prior to the experiment that the person in video is a young adult male (approximately 24 years of age) without any neurological issues. The order in which the judgment tasks were completed was counter-balanced across participants.

After the participants completed both judgment tasks, they completed an execution task in which they completed reciprocal aiming movements to the targets on each of the nine boards. Participants started with the index finger of their right hand on the right target and were asked to complete 10 movements between the targets as fast as possible without missing the target. Two trials of 10 movements were completed on each target arrangement. If the participant completed more or less than 10 movements, the calculation of the average MT was adjusted accordingly (see below). The order in which the target contexts were presented was randomized for each participant. The movements were recorded with an active marker motion capture system (Optotrak 3020, Northern Digital Inc.). A single infrared light emitting diode (IRED) was taped to the tip of the index finger of the participant. The location of the IRED was recorded at a rate of 200 Hz for a period of 15 s and stored on a computer for offline analysis.

### Data Reduction and Analyses

The apparent MTs for the judgment tasks were computed as the SOA between the two images when the “Enter” button was pressed. To determine MT for the execution task, a custom analysis program written in Matlab identified the interval of time from the start to the end of the movement sequence. The raw displacement data were differentiated using a 3-point central finite algorithm to obtain instantaneous velocity values. The start and end of the movement sequence were identified as the first sample in which velocity in the primary axis of movement (left/right or × axis) was below or above than 30 mm/s for 100 ms, respectively. Average MT was calculated by dividing the length of time of the movement sequence by the number of completed movements. No movements landed outside of the target boundaries and so no individual MTs were eliminated due to aiming errors. Three trials were deleted from the data set due to identified recording errors.

Mean MTs for the different combinations of target width and movement amplitude were calculated for each individual and were submitted to a series of specific analyses to address the experimental hypotheses. Prior to conducting these analyses, all data for one participant in the below cervical SCI group was eliminated because all MTs of that individual in the execution task were more than 3 standard deviations above the mean MTs of the participants in that group. All MTs for the participants within the cervical group were within 2 standard deviations of the mean for that group. Two separate independent sample t-tests comparing the ages and years since injury revealed that there were no significant differences between the final groups of participants in these characteristics – age, *t*(19)  = 1.05, *p*>0.3; years since injury, *t*(19)  = 0.59, *p*>0.5). Even though there were no group differences in these factors, the potential influence of age or time since injury on the pattern of results was assessed through initial analyses wherein these factors were used as covariates. The results of these analyses did not reveal any meaningful changes in the effects as reported below in the Results section. As such, the results of the analyses without these factors as covariates are reported.

A series of planned comparisons were conducted to test the experimental hypotheses. The initial analysis consisted of a series of linear regressions between the mean MTs for each group and each of the 9 combinations of target width and movement amplitude (ID 2, 3, and 4). The purpose of these initial analyses was to determine if the MTs in each of the tasks conformed to Fitts's Law. The second analysis tested whether or not the groups differed in upper-limb performance capabilities. To this end, mean MTs averaged across the different combinations of distance and width for a given ID for each individual on the execution task were submitted to a 2 (Group: below cervical, cervical) by 3 (ID: 2, 3, 4) mixed ANOVAs with Group as a between-subject factor and ID as repeated measures factor. Subsequently, a correlational analysis between self-perceived arm function rating and movement times (averaged across all target distance and width combinations) in the execution task. The purpose of this analysis was to assess the reliability of the self-perceived arm rating scale (see Table 1). As stated above, participants were sorted based on the level of injury, but were asked prior to their participation about their level of perceived function. Although the most theoretically-relevant index of motor function for the present purposes would be MTs on the execution task because this is an actual measure of their performance on the movement task, it was also of interest to determine and confirm that each individual's perception of their own general function relates to the performed MTs.

The third and most theoretically-relevant analysis tested the main experimental hypothesis regarding the action possibility judgments. Mean MTs averaged across the different combinations of distance and width for a given ID for each individual from the different judgment tasks were submitted to 2 (Group (below cervical, cervical) by 2 (Task: self-, other-judgment) by 3 (ID: 2, 3, 4) mixed ANOVA with Group as a between-subject factor and Task and ID as repeated measures factor. Post hoc testing of all significant effects involving more than 2 means was conducting using Tukey's *HSD*. As with the analysis of actual execution MTs, mean (averaged across all different combinations of distance and widths) MTs on the self- and other-perception tasks were correlated with the 1–4 rating scores on the self-perceived arm rating scale (see Table 1) to determine if each individuals' perception of their own general function relates to the perception of their MTs and those of the person in the video. Alpha was set at 0.05 for all tests outlined to this point and all significant differences are reported.

Finally, a series of correlational analyses were conducted to assess the relationships between the MTs on the judgment tasks and the execution task on the individual participant level. The rationale for these analyses was that if formation of the judgments were based on a simulation process that uses networks that are responsible for generating the actions and coding the perceptual consequences of the actions, then the characteristics of the times across judgment and execution tasks should be similar. If, on the other hand, different processes underlie the judgment and execution tasks, then there should not be any similarities among the MTs of the tasks. Based on this rationale, the first analysis involved a series of 3 correlations in which the relation between each individual's mean MT (averaged across all combinations of target distance and amplitude) for each of the tasks was assessed. In the final set of analyses, regression lines for the relation between MT and index of difficulty for each participant was calculated for each of the 3 tasks. The slopes and y-intercepts of the regressions lines were compared to one another to determine if these components differed across the tasks (for details on the statistical procedure, see Chapter 18 of [14]). This approach has previously been employed to investigate whether or not there are similarities in the characteristics of an individual's performance on perception, imagination, and execution tasks [8]. Due to the large number of comparisons in this particular set of analyses, alpha was corrected to 0.00238 (0.05/21 – the number of participants in the study and, as a result, the number of comparisons for each between-task comparison for each component). Note also that, for the sake of brevity, the results of each comparison and the details of each regression line are not being reported here. Instead, only summary data and tallies of the numbers of significantly different slopes and y-intercepts are reported.

## Results

### Movement Times as a Function of Index of Difficulty

The results of the linear regression analyses revealed that MTs for each group conformed to Fitts's Law in each task. Results of the analyses are as follows: cervical group – self-judgment, MT = 313+62(ID), R^2^ = .55, *p*<.05; other-judgment, MT = 212+36(ID), R^2^ = .67, *p*<.05; execution, MT = 177+64(ID), R^2^ = .67, *p*<.05; below cervical group - self-judgment, MT = 178+47(ID), R^2^ = .76, *p*<.05; other-judgment, MT = 133+40(ID), R^2^ = .74, *p*<.05; execution, MT = 141+44(ID), R^2^ = .66, *p*<.05. Because the data for each group and condition conformed to Fitts's Law, the data for the individual combinations of target width and movement amplitude were averaged within an ID to generated average MTs for each ID for each analysis in which ID was a factor (see [5]).

### Movement Times in the Execution Task

**Figure 2:**
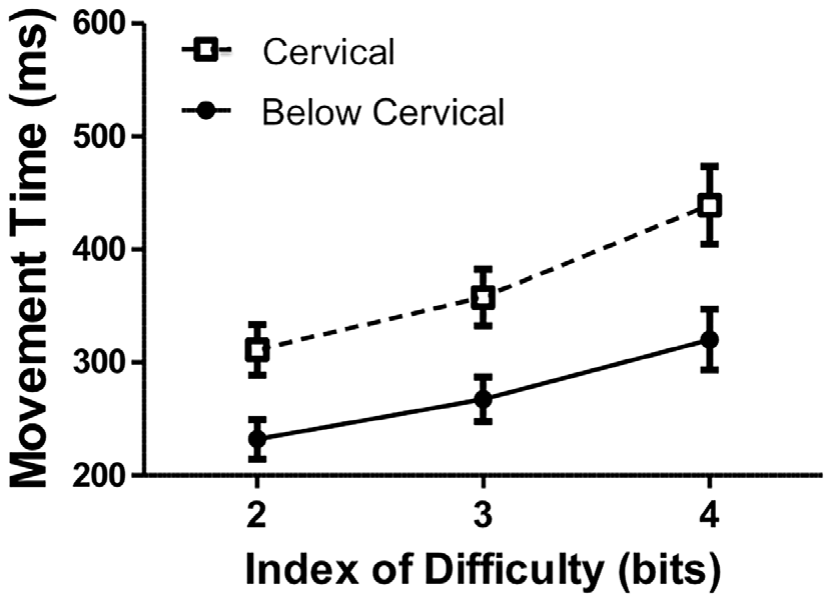
Mean movement times (ms) as a function of Group and Index of Difficulty for the execution task. Open symbols with dashed connecting lines represent data from the group with cervical SCI. Closed symbols with solid connecting lines represent data from the group with below cervical SCI. SEM bars are shown.

The analysis of MTs from the execution task revealed main effects of ID, *F*(2, 38)  = 79.76, *p*<.05, and Group, *F*(1, 19)  = 8.08, *p*<.05. The Group by ID interaction approached but did not reach significance, *F*(2, 38)  = 2.81, *p>*.05. Overall, MTs increased with increases in movement difficulty as predicted by Fitts's Law (ID 2 = 272 ms; ID 3 = 312 ms; ID 4 = 380 ms). Further, the mean overall MTs for the group with cervical level injuries (369 ms) were longer than those for the group with below cervical SCI injuries (273 ms) (see Figure 2). For the correlation analyses, the categories of perceived arm function (see Table 1) were converted into a 1–4 Likert scale (1 =  Below Functional to 4 =  Very Good). Each participants' (i.e., participants in both the cervical and below cervical groups) assessment of their general capabilities were then correlated with movement times in the execution task. As expected, perceived arm function was significantly negatively correlated with movement times in the performance task, *r* = −.57, *p*<.01. The results of this analysis indicate that mean MTs decreased as the participants reported arm function increased. Although the observed between-group differences in MTs on the actual movement task is the most theoretically-relevant index of motor capability for the present purposes, it is reassuring to observe that the participant's assessment of their own capabilities on a 1–4 scale is consistent with their actual performance on the present movement task.

### Movement Times in the Judgment Tasks

**Figure 3:**
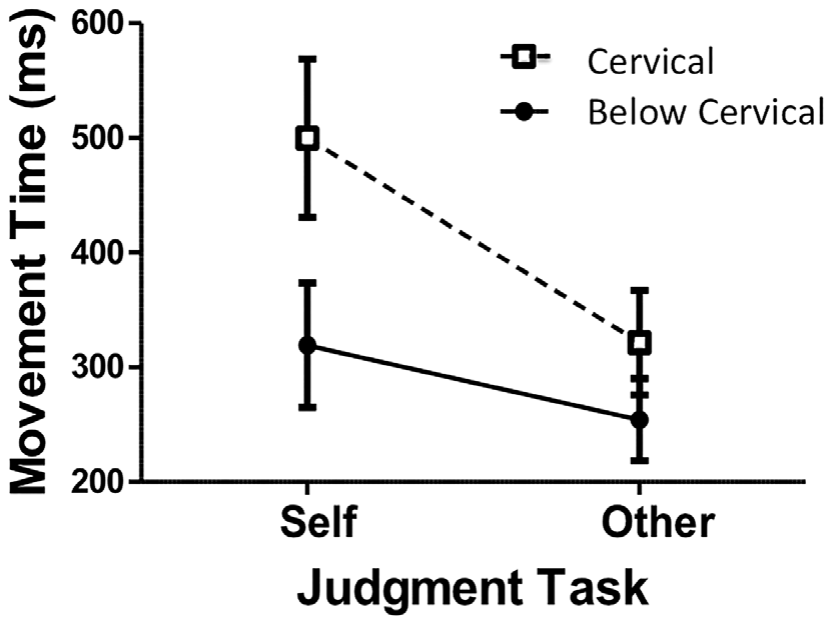
Mean movement times (ms) as a function of Group and Judgment Task. Open symbols with dashed connecting lines represent data from the group with cervical SCI. Closed symbols with solid connecting lines represent data from the group with below cervical SCI. SEM bars are shown.

Consistent with the analysis of execution MTs, there was a significant main effect for ID in judgment MTs, *F*(2, 38)  = 18.75, *p*<.05, indicating that MTs increased with increasing ID (ID 2 = 303 ms; ID 3 = 345 ms; ID 4 = 396 ms). There was also a main effect for Task, *F*(1, 19)  = 24.64, *p*<.05, revealing that MTs in the self-judgment task (409 ms) were longer than those on the other-judgment task (287 ms). Although the main effect for Group was not significant, *F*(1, 19)  = 3.11, *p*>.05, the interaction between Group and Task was significant, *F*(1, 19)  = 5.35, *p*<.05. Post hoc analysis of this interaction revealed that the MTs for the group with cervical SCI in the self-judgment task were longer than all other MTs, and that all other MTs did not differ (see Figure 3). That is, there were no statistical differences in the MTs in the self- and other-judgment tasks for the group with below cervical SCI suggesting that, consistent with their level of injury, they judged that they would perform the task with similar MTs to an individual without any neurological issues. Further, similar to actual performance differences (see preceding analysis of execution MTs), the shortest MTs at which the group with cervical SCI judged that they could perform the task were longer than the shortest MTs at which the group with below cervical SCI thought they could complete the task. Relatedly (but perhaps of greater theoretical relevance), there were significantly shorter MTs in the other-judgment than in the self-judgment task for the group with cervical SCI. This result indicates that the individuals with cervical SCI adapted their judgment to the assumed capabilities of the neuro-typical individual. Finally, there were no reliable between-group differences in the MTs for the other-judgment task suggesting that the two groups judged the individual without neurological issues to be able to perform the movement task with similar capabilities.

The results of the subsequent correlation analyses in which the relationship between each participant's (i.e., participants in both the cervical and below cervical groups) perceived arm function and MTs on the self- and other-judgment task were consistent with the results of the analysis. That is, perceived arm function was significantly negatively correlated with MTs in the self-perception task, *r* = −.62, p<0.01. This finding indicates that, consistent with their actual performance (see preceding Section), participants judgments of their motor performance in the aiming task were consistent with their general assessment of their own abilities. The correlation between perceived arm function and the judgments of the other persons capabilities approached, but did not surpass, conventional levels of significance, *r* = −43, *p*>0.05, suggesting that their assessments of other people's capabilities may be influenced by, but was not completely determined by, their own perception of their general functional capabilities.

### Relationships among Movement Times in each Task

The analyses of mean MTs in each of the tasks revealed significant correlations between the MTs in each of the tasks. Specifically, mean execution MTs were significantly correlated with mean MTs on the self-judgment task, *r* = 0.67, *p*<.001, and the other-judgment task, *r* = 0.51, *p*<.05. Mean MTs on the two judgment tasks were also significantly correlated, *r* = 0.85, *p*<.001. Although the correlation between execution and self-judgment MTs was slightly stronger than between execution and other-judgment MTs, this difference was not significant, Steiger's *Z* = 1.64, *p*>0.05. In sum, the results of this analysis are consistent with the notion that there are similar processes underlying each of the tasks.

The results of the final set of analyses are consistent with what was previously observed [8] and the hypothesis that a simulation of an individual's own performance is an important component of the judgment process for a specific task. Specifically, the comparisons between the components of each participant's regression lines of the execution and self-judgment tasks revealed that the slopes of only 2 of the 21 participants differed (both differences were observed for participants in the group with below cervical SCI). In contrast, comparisons of the y-intercepts of each individual's regression line revealed significant differences for 8 of the 21 participants (6 of the 13 participants with below cervical SCI and 2 of the 8 with cervical SCI). This overall consistency in the slopes and individual variability in the y-intercepts for execution and self-judgment has been observed previously [8].

Comparisons of the slopes of the regression lines between MT and ID for the execution and other-judgment tasks for each individual did not reveal any significant differences. The y-intercepts of the regression lines of the two tasks, on the other hand, were different for 10 participants. Interestingly, unlike for the comparisons of execution and self-judgment in which differences in y-intercepts was only observed for 2 of 8 people in the cervical SCI group, the y-intercepts of the lines for execution and other-judgment task were significantly different for a majority (5/8) of the participants with cervical SCI. Y-intercepts were significantly different for only 5 of the 13 participants with below cervical SCI.

Finally, comparisons of the regression lines between MT and ID for the self-judgment and other-judgment tasks did not reveal any between-task differences in the slopes. The y-intercepts of the two tasks were different for 8 participants. Again, the differences in y-intercepts occurred for the majority of participants with cervical SCI (6 of 8), whereas only different for 2 of the 13 participants with below SCI had significant differences.

Overall, the results of these correlation analyses are consistent with the results of the ANOVAs reported in preceding sections. Specifically, these analyses revealed that: 1) each participants' judgment of their own performance was generally consistent with their actual performance; 2) the judgments of the participants with below cervical SCI of their own performance was generally not different from their judgments of what the young adult model could perform; and 3) the majority of the participants with cervical SCI judged their performance to be different from what the model in the video could perform. This latter difference was also expressed in comparisons between the participants with cervical SCI actual performance and the judgments of what the other person could perform. In addition, the results of these analyses are consistent with the hypothesis that a common process or network underlies the execution and judgment of action because the slopes of the regression lines for each task rarely differed (i.e., only 2 of 63 comparisons revealed significantly different slopes across the tasks). As will be addressed in greater detail in the Discussion section (see also [8]), this overall pattern of effects suggests that the judgments are largely based on a simulation of the participants own performance and that judgments for other people are adapted by shifting the criterion of what is possible by a certain amount.

## Discussion

The present study was conducted to investigate the formation of action possibility judgments by people with different motor capabilities due to SCI. Participants with cervical or below cervical SCI were asked to judge the shortest MT at which it was possible for themselves or another person (young adult male without neurological issues) to complete different aiming movements accurately. To assess the participants' actual motor performance on the perception tasks, each participant also executed the movements. It was found that the MTs of the group with cervical SCI were longer than those from the group with below cervical SCI in both the self-judgment and execution tasks. This result indicates that the participants' judgments of their own capabilities are consistent with relative performance differences (see [5]). The most interesting pattern of findings in the present study, however, was that the MT judgments of the other person's capabilities formed by the group with cervical SCI were significantly shorter than judgments of their own capabilities, and were not reliably different from the judgments made by the group with below cervical SCI. These findings suggest that people with SCI are able to effectively adapt their judgments and, hence, that the judgments of action possibilities are not completely bound to one's own current movement capabilities. Overall, this present pattern of findings provides new insight into the processes underlying action possibility judgments. The following discussion will begin with a consideration of the relationship between an individual's own motor capabilities and judgments, and then turn to the neuro-cognitive processes that lead to the formation of judgments about other people's movement capabilities.

The first finding of note was that the participants with cervical SCI had longer MTs in the self-judgment task than the group with below cervical SCI. These differences cannot be attributed to age or any number of factors associated with SCI in general because there were no group differences in these factors. Instead, the between-group difference in the self-judgment task can be attributed to the relatively accurate prediction of what the individuals are able to perform. Note this difference was also found when comparing execution MTs for both groups. These data are consistent with previous research on the relationship between movement capabilities and action judgments in this aiming task. For example, Eskenazi et al. [12] reported that neither the performance nor the judgment MTs of a person with a frontal lesion was affected by target width. Further, Chandrasekharan et al. [1] demonstrated that: 1) experience with the task improved action possibility judgments; and, 2) that the judgments were affected by the wearing of a weight. These results suggest that predictions of MT are shaped by the motor capabilities of the person forming the judgments. Taken together, these findings support the hypothesis that action possibility judgments are based on an internal simulation using one's own motor capabilities [2], [5], [8].

Of greater theoretical relevance to the present study, however, is the pattern of findings that emerged via comparisons of MTs on both judgment tasks. Specifically, there were no between-group differences in judgments for what the young adult male could perform. The similarity in MTs in the judgments for the other person emerged because the group with cervical SCI adjusted their judgments (i.e., there were significantly shorter MTs in the other-judgments than in the self-judgments). This result is relevant for at least two reasons. First, these findings replicate and extend previous research on the adaptability of the judgment process during the formation of judgments for different individuals (e.g., [5], [15], [16]).

Second, the present data make a novel contribution to the literature because they reveal that people with limb-specific movement deficits can adapt their judgments in a manner consistent with people with intact limb function. On the one hand, the present findings may not be too surprising because, although individuals with cervical SCI have altered motor capabilities, the CNS damage leading to the performance differences involves spinal circuitry. The super-segmental systems that are likely essential to the judgment process remain intact and, hence, the judgment process should likewise remain intact. On the other hand, the data presented here provide an interesting contrast to those of Bosbach et al. [13] who observed that individuals who have lost proprioceptive and haptic information due to a peripheral sensory neuropathy showed a deficit in estimating the weight of an object a model was lifting based on movement kinematics. Because one of the individuals was able to successfully lift one set of the objects (i.e., had relatively intact motor processes), Bosbach et al. concluded that the deficit in weight estimation via action observation occurred because the action-perception networks (i.e., the internal model) underlying the simulation process may have decayed as the result of prolonged loss of sensory input [13]. Recall that the individuals with cervical SCI in the present study had partial upper-limb motor and sensory function. Thus, it is perhaps because of the partial motor function and/or sensory input that the present participants were able to maintain the integrity of their pre-existing (i.e., pre-injury) action-perception networks (see later paragraphs for more discussion). The results of the present study make it clear, however, that people with partial sensorimotor limb function are able to form judgments about what is possible for themselves and others to perform. This leads one to wonder whether or not people with complete sensorimotor quadriplegia can form and adapt action possibility judgments for people with varying abilities (i.e., as in [5]). Such a pertinent question is beyond the scope of the present study because inclusion criteria was the ability to perform the task, and the variation in time since injury so relative differences in the judgment tasks can be rectified to differences in execution.

To return to the main research question of the present paper, the results of the present and other [5], [8] experiments reveal that action possibility judgments are not completely dependent on an individual's present motor capabilities. As discussed in the Introduction, it has been suggested that a simulation of the individuals' own movement performance likely forms the core of the action possibility judgment process [1], [2], [8]. The data reported here and elsewhere (e.g., [5]) suggest that this simulation is not the sole process, otherwise such context- and/or person-dependent adaptations would not be observed. Although these data reveal that people can adapt their judgments, it is not possible to definitively determine if the adaptation was enacted via the modulation of the simulation process or of the threshold for what is possible or impossible to perform. Based largely on the results of the comparisons of the slope and regression lines of MT and ID for each individual, it is suggested here that it is likely the threshold for the decision is adjusted during the judgment process (see also [5], [8]). Before providing a full discussion of this explanation, another potential explanation for how individuals with cervical SCI formed their judgments for the person without an SCI will be considered.

One account of the processes leading to the adapted MTs observed in the present study is that the individuals with cervical SCI engage internal models or perception/action coding systems, and use pre-injury (i.e., fully functional) networks to access passed movement capabilities, and generate a simulation of the performance of the young adult in the video. Some support for this possibility might be drawn from the literature examining brain activation in attempted action and action imagery in people with SCI. For example, Hotz-Boendermaker et al. [17] sought to determine if individuals with paraplegia demonstrated different levels and patterns of neural activation from their neuro-typical peers in attempted and imagined foot movements. In this study, both control and SCI patients completed imagined (or simulated) movements and executed (or attempted to execute movements in the case of SCI patients) movements as well. During each of the tasks, brain activation patterns were assessed by fMRI. Overall, the researchers found that both people with SCI and control subjects displayed activation in separate networks for imagined and executed movements. Also, very similar networks were activated for both groups in both tasks. That is, when participants with SCI simulated or attempted to execute their movements, the networks activated in each case were similar to the networks activated in the analogous tasks for healthy controls. These findings led the researchers to conclude that the central motor programs for these movements may be preserved. Similar findings were noted throughout the literature for a variety of tasks and imaging protocols [18], [19]. The general conclusion arising from this body of work is that motor programs for previous real movements may remain intact in individuals with SCI regardless of degree of actual limb function (cf., [13]). Thus, it is possible that the simulation used in the judgment process investigated in the present study engaged preserved pre-injury networks.

The preferred explanation for the present data, however, is that the participants simulated their own performance of the task and then adjusted their threshold for what is possible for someone else to perform by adapting the threshold for what is possible or impossible based on the assumed difference [5]. The main evidence in support of this explanation is derived from the last series of correlational analyses in which the slopes and y-intercepts of the regression lines for MT and ID for each individual were compared across each of the tasks. The results of this analysis revealed that any significant between-task differences in the components that emerged were in the comparisons of the y-intercepts (baseline MT); with the majority of these differences emerging for the group with cervical SCI occurring in the self/other and execution/other comparisons. The slopes of the lines (which reflect the increase in MT for an increase in ID) only differed on 2 of the 63 comparisons and never differed for any of the comparisons involving the group of individuals with cervical SCI. It is argued here that this pattern of effects suggests that participants formed their judgments by simulating their own performance of the task conditions they were observing on the screen and then adjusted their judgment of what is possible by changing the threshold for what is possible for the other person to perform by a constant amount based on that the estimated difference in performance capabilities would be (see also [5]). To understand how the patterns of data are likely to be an expression of this process, an account of what might happen on each trial is required.

On each instance in which a judgment is required (i.e., each trial), the individual simulates their own performance of the task and, as a result, determines what it is possible for themselves. As an example, a participant might observe the model moving in a target condition in which the ID is 3 and, through a simulation of their performance, determine that they are able to complete the movements with a MT of 400 ms. If the participant in this example estimates that the other individual can perform the task 100 ms faster, then they would subsequently adjust their threshold for possible/impossible down to 300 ms. On the following trial, the participant might observe the model moving in a target condition in which the ID is 4, and simulate and judge their own performance capability to be at 450 ms. Adjusting the threshold for the judgment for the other person down by 100 ms would yield a MT of 350. Over repeated trials with different index of difficulties, these adjusted judgments for each individual trial form a new regression line for what the other person can do that has the same slope as the line characterizing the person's own relationship between MT and ID, but that has a different y-intercept depending on if the person forming the judgments estimates that the other individual is better or poorer performer. This exact pattern of similar slopes and different y-intercepts was observed in the present data (see also [5]). Thus, it is proposed here that the present correlational analyses indicate that, although the participants with cervical SCI still engage a simulation of their own performance during the formation of the action possibility judgments for other people (as revealed by the similarity in slopes across all tasks). Thus, it is evident that the simulation process does not entirely bias their judgment of what other people can do and individuals are able to effectively adjust their judgment to account for estimated differences in performance. In the end, the nature of the adaptation process and the types of networks used during the simulations is an open question and will require additional research to answer.

## Conclusions

The ability to judgment the action capabilities of different individuals is an important process for the completion of joint actions. The core process involved in determining what is and is not able to be perform involves a simulation of one's own action utilizing the brain's linked perception and action networks. The present study examined if changes in motor capabilities due to different levels of spinal cord injury would lead to alterations in the judgment process. The results suggest that the judgment process is intact in persons with motor deficits due to SCI. Future studies will need to clarify the nature of the simulation process in people with SCI.
